# Large-scale health system transformation in the United Kingdom: Implementing the new care models in the NHS

**DOI:** 10.1108/JHOM-05-2019-0144

**Published:** 2020-03-17

**Authors:** Gregory Maniatopoulos, David J. Hunter, Jonathan Erskine, Bob Hudson

**Affiliations:** 1 Institute of Health and Society, Newcastle University, Newcastle upon Tyne, UK; 2 Durham UniversityUK; 3 Centre for Health Services Studies, Univeristy of Kent, Kent, UK

**Keywords:** National health service, England, Implementation, Health systems, Large-scale transformation, New care models

## Abstract

**Purpose:**

Following publication of a new vision for the English National Health Service (NHS) in 2014, known as the NHS Five-Year Forward View, a Vanguard programme was introduced by NHS England charged with the task of designing and delivering a range of new care models (NCMs) aimed at tackling deep-seated problems of a type facing all health systems to a greater or lesser degree. Drawing upon recent theoretical developments on the multilevel nature of context, we explore factors shaping the implementation of five NCM initiatives in the North East of England.

**Design/methodology/approach:**

Data collection was based on semi-structured interviews (66 in total) between December 2016 and May 2017 with key informants at each site and a detailed review of Trusts' internal documents and policies related to the implementation of each NCM. Our analysis explores factors shaping the implementation of five NCM pilot sites as they touched on the multiple levels of context ranging from the macro policy level to the micro-level setting of workforce redesign.

**Findings:**

It is far too early to conclude with any confidence that a successful outcome for the NCM programme will be forthcoming although the NHS Long-Term Plan seeks to build on the earlier vision set out in the Five-Year Forward View. Early indications show some signs of promise, especially where there is evidence of the ground having been prepared and changes already being put in place prior to the official launch of NCM initiatives. At the same time our findings demonstrate that all five pilot sites experienced, and were subject to, unrealistic pressure placed upon them to deliver outcomes.

**Originality/value:**

Our findings demonstrate the need for a deeper understanding of the multilevel nature of context by exploring factors shaping the implementation of five NCMs in the North East of England. Exploring the wider national policy context is desirable as well as understanding the perceptions of front-line staff and service users in order to establish the degree of alignment or, conversely, to identify where policy and practice are at risk of pushing and pulling against each other.

## Introduction

With health systems globally facing new and complex challenges, transforming the way services are organised and provided to meet rapidly changing needs has become a major preoccupation of, and priority for, policy-makers with increasing attention being given to improving population health and strengthening integrated care ( Hunter *et al.* , 2015 ; WHO, 2016 , 2018 ). In the United Kingdom (UK), the health and social care system is facing mounting pressures aiming to improve outcomes and reduce inequalities at a time of increasing financial stringency and uncertainty. In particular, recent epidemiological and demographic changes, notably an aging population and a rise in co-morbidities, coupled with technological innovation and new treatments, have stimulated the appetite for new approaches and ways of working directed towards improving population health, quality and patient safety ( Rechel *et al.* , 2009 ). In this context, developing new models of care and related transformation programmes as reflected in an ambitious vision for the English National Health Service (NHS) “to create the biggest national move to integrated care of any major western country”, have become central themes in health policy ( NHS England, 2017 ). Similar moves are underway in the three other countries making up the United Kingdom but are not our concern in this paper.

Following publication of the *NHS Five-Year Forward View* (5YFV) in 2014, a Vanguard programme was introduced by NHS England (the executive non-departmental public body of the Department of Health and Social Care which oversees the NHS) to test different approaches to health and social care service delivery ( NHS England, 2017 ). These reform initiatives have typically taken place under the banner of Triple Aim thinking with its focus on population health, effective patient-centred care and per capita cost ( Berwick *et al.* , 2008 ). The NHS invited individual organisations and partnerships, including those with voluntary and community sector involvement, to apply to become pilot sites for the new care models (NCMs) programme. Overall, 50 pilot sites were established across England charged with the task of designing and delivering a range of NCMs aimed at tackling deep-seated problems of a type facing all health systems to a greater or lesser degree. The NCMs include: managing rising demand on accident and emergency services, keeping people out of hospital, effecting rapid discharge for those no longer in need of acute care, integrating health and social care, reducing silo working and giving higher priority to prevention. These new models of care proposed changes that are not concerned solely with structures or top-down reform edicts but are seeking new ways of achieving integrated care and joined up working across a whole system, driven by those on the front-line displaying new behaviours and leadership styles with an emphasis on collaboration rather than competition. In so doing, the overall aim of the NCMs programme was to encourage the adoption of a whole system focus on health and well-being rather than ill-health and disease and to strengthen integration across health and social care.

Against this background, the paper explores factors shaping the implementation of five NCM initiatives in the North East region of England ( Maniatopoulos *et al.* , 2017 ). It draws upon a study, conducted over 12 months, which explored the implementation arrangements of the following NCMs: Multispecialty Community Providers (MCP); Integrated Primary and Acute Care Systems (PACS); Acute Care Collaboration (ACC) Enhanced Health in Care Homes (EHCH) ; and Urgent and Emergency Care (UEC) (see Table AI for a brief description of each NCM). In line with policy pressures to move resources out of hospital care, these pilots aimed to reconfigure the way health care is organised and delivered by shifting care from acute hospitals to primary or community-based health services and by strengthening health and social care integration. The reported study was conducted during a time of ongoing policy changes in the NHS, notably developments surrounding integrated policy frameworks such as Sustainability and Transformation Partnerships (STPs), Accountable Care Organisations (ACOs) and Accountable Care Systems (ACSs) which aimed to promote whole system, place-based collaboration to enable the delivery of integrated care and joined up working across health and social care.

In seeking to understand the changes and the likelihood of success, we draw upon Pettigrew *et al.* ’s ( 1992a , b ) “receptive contexts for change” framework, which we combine with more recent theoretical developments aiming to address the multilevel nature of context ( Maniatopoulos *et al.* , 2015 ; Greenhalgh *et al.* , 2017 ). We consider that the framework remains valid for explaining and understanding organisational change and the reasons for variations across different settings. But it can also benefit from revisiting in the light of more recent analyses exploring the multiple levels of context (macro, meso and micro) shaping implementation and change which have gained currency amongst organisational and healthcare researchers. Our purpose here is to broaden the scope and scale of analysis across the multiple levels of context shaping both process and outcomes of health systems transformation. In so doing, we aim to contribute to the study of the multilevel nature of context by highlighting the political context of policy implementation at both the macro and micro levels. Previous implementation studies of complex public policies involving diverse and unequal partners have failed to locate the policy in the macro political and ideological context as well in the micro level of everyday practice ( Hunter *et al.* , 2015 ; Maniatopoulos *et al.* , 2015 ).

The paper is organised as follows. The next section summarises the current policy developments around health and social care transformation in England. Following this discussion, we explore Pettigrew *et al.* ’s ( 1992a , b ) “receptive contexts for change” framework which seeks to identify the key factors shaping implementation in large-scale change initiatives occurring in complex settings. We then move to consider more recent theoretical developments which seek to address the multilevel nature of context. Subsequent sections describe the methodology and the context of the study before presenting and discussing its main findings. The paper concludes with a review of the key issues that are common across all five NCMs, drawing out any emerging lessons to be learned from the implementation with a view to informing future transformational change underway more widely in the NHS.

## The policy landscape on health system transformation: implications for the English NHS

Health system transformation is a feature of health systems globally. The challenges facing such systems comprise the third era of healthcare transformation ( Halfon *et al.* , 2014 ) with its emphasis on integrated care, population health and a place-based approach to joining up services around communities and individuals in need of support. The NHS is no exception. In line with global health policy developments, the government has embarked on an ambitious strategy to transform the NHS in England from a reactive “sick” largely hospital care service to a proactive health system ( Wanless, 2004 ). The disease burden from avoidable lifestyle-related diseases is around 86 per cent as estimated by WHO Europe ( WHO, 2012 ). In order to survive as a publicly funded system of health care, current UK policy emphasises the need for a higher priority to be accorded to prevention and public health and to strengthen integrated care across the health and social care interface.

The Health and Social Care Act (HSCA) 2012 in England which is the legislative basis for the current NHS structure led to significant changes in the organisation of the NHS. It has proved extremely unpopular with the health care professions and others principally because it has led to a competitive, fragmented system of care which has made meeting the challenges noted earlier more difficult ( Walshe, 2014 ; Hunter, 2016 ). In order to avoid new legislation, which is unlikely to be forthcoming given the government's all-consuming preoccupation with leaving the European Union following the referendum in 2016, and further structural change for which there is little appetite in government, a different approach to change was adopted. This emphasised the need to move away from a system that encourages competition among providers (both primary and acute care) and acknowledged the complexity of health systems and the need to empower the front line to lead on implementing changes. The vision set out in the *5YFV* provided the framework for such a shift with its emphasis on public health, integrated care and exploring new models of meeting changing health care needs in response to diverse local circumstances. The NCMs being implemented across the country were a prelude to more ambitious initiatives including the development of STPs and, more recently, ICSs aiming to bring together NHS organisations and their partners to plan and oversee the implementation of improvements in health and care by focusing on local system partnerships rather than isolated activity undertaken by any single organisation. Examples of the changes underway in some areas include the development of more strategic approaches to health and social care commissioning and the emergence of partnership work between community providers.

With the publication of the NHS Long-Term Plan in early 2019, further developments with Integrated Care Partnerships (ICPs) and place-based approaches to health and well-being are proposed ( NHS England, 2019 ). The contemporary policy landscape therefore remains fluid and unstable so the endgame is presently unknown. There are also calls for new legislation in order to establish with greater clarity what the new governance arrangements might look like and to establish where accountability lies for the changes that have been set in train especially when these require engagement from local government if they are to succeed.

## Implementation, context and change: towards a multilevel contextual analysis

In recent years, considerable attention has been given to understanding the vital role of context when implementing change initiatives in healthcare systems ( Dopson and Fitzgerald, 2005 ; Kaplan *et al.* , 2010 ; Aarons *et al.* , 2011 ; Bate, 2014 ; Greener *et al.* , 2014 ; Fulop and Robert, 2015 ; Squires *et al.* , 2015 ; May *et al.* , 2016 ). Theory and research addressing the diffusion of changes in healthcare organisations have accelerated and are pursued across a large, diverse and complex literature and related frameworks and disciplines which seek to explore the contextual factors shaping the implementation process ( Greenhalgh *et al.* , 2004 , 2005 ; Kyratsis *et al.* , 2012 ; Nielsen, 2015 ). There are various conceptualisations of context which reflect the variety of different perspectives exploring the recursive relationship between human action and the wider organisational and system context ( Greenhalgh *et al.* , 2016 ; Damschroder *et al.* , 2009 ). A key distinction that contextual perspectives recognise is between “inner” (immediate, intra-organisational) and “outer” (social, political) contexts which can vary considerably between organisations ( Shaw *et al.* , 2017 ).

Pettigrew *et al.* ’s ( 1992a , b ) “receptive contexts for change” framework was one of the first attempts to explicitly recognise the complex, multi-faceted nature of implementing changes in practice. Such a perspective challenges the conventional split between policy formulation and implementation by viewing these processes not as discrete but as interactive and muddled ( Pettigrew, 1990 ; Pettigrew *et al.* , 1992a ). The basic proposition of Pettigrew *et al.* ’s framework is that any analysis of change should focus not solely on the content of the change initiative but also on the process (including actions and interactions of key players) and on the context (both local or “inner” context and the “outer” context of national and regional policies and events) ( Pettigrew *et al.* , 1992a , b ). This requires viewing change as a multi-faceted process, involving social, political, cultural, environmental and structural, as well as rational dimensions. Receptive contexts are defined as situations where there are features of context, and also of individual action, that seem to be favourably disposed to change and are associated with forward movement. Conversely, non-receptive contexts are those situations where a combination of conditions effectively creates blockages or resistance to change. The key factors comprising the “receptive contexts for change” framework are summarised in Table I .

While Pettigrew *et al.* ’s ( 1992a , b ) framework highlighted the complex, multi-faceted nature of implementing changes, we argue that it can be strengthened by being combined with more contemporary perspectives which pay more explicit attention to the multiple levels of context (macro, meso and micro) and, crucially, to how reconfigurations across a multilevel set of practices shape implementation and change. Recent theoretical developments have attempted to explore further the interdependent relationships between different structural elements of the context across a multilevel set of practices ( Maniatopoulos *et al.* , 2015 ; Greenhalgh *et al.* , 2017 , 2016 ; Robert *et al.* , 2010 ). Such perspectives recognise the mediating role of context, not just at the immediate level of implementation but at the policy, systems and organisational levels, where the complexities associated with the political economy of healthcare (e.g. funding and commissioning) are important determinants to the success, or failure, of implementation ( Maniatopoulos *et al.* , 2015 ). This focus on the local and more distant social, political and economic influences has a key role in the so-called theories of social practice which aim to produce rich theorisations of the process of implementation as an outcome of social practice ( Greenhalgh *et al.* , 2017 ). Drawing upon wider intellectual resources, such approaches aim to move away from the view of context as a layered and unidirectional set of influences with a pre-existing “top” and “bottom” structures by highlighting the dynamic nature of context, that is to say, context is seen as evolving and changing over time ( Maniatopoulos *et al.* , 2015 ; Dopson and Fitzgerald, 2005 ). Accordingly, the boundary between inner and outer context far from being given and/or fixed becomes both socially configured and reconfigurable, thus allowing alternative ways of reshaping organisational change. From this perspective, implementation is seen as an emergent and contingent process of contextual and relational organising through “sense-making” ( Weick, 1995 ; Peck and 6, 2006 ). From a health policy context, such an approach reinforces previous attempts to reassess models of policy implementation in the “congested state” notable for multilevel governance and a need to align both vertical (centre–local) and horizontal (central–central and local–local) axes ( Exworthy and Powell, 2004 ). Drawing upon the multilevel nature of context (macro, meso and micro), we explore factors shaping the implementation of five NCM initiatives in the North East region of England.

## Methods

The study (NHS funded) aimed to identify factors shaping the implementation of the NCMs programme in the North East of England. Data collection was based on semi-structured interviews (66 in total; see Table I ) between December 2016 and May 2017 with key informants at each site and a detailed review of Trusts' internal documents and policies related to the implementation of each NCM. Following ethical approval from Newcastle University Ethics Committee (ref: 01216/2016), participants were purposively selected according to their role and involvement in the implementation of each NCM and included clinicians, chief executives, commissioner managers, project managers and other specialists. Participants were provided with information sheets in advance and consent forms signed prior to the start of the interviews. Interviews lasted around one hour and were digitally recorded and transcribed (see Table II ).

A topic guide, informed by published literature on health systems transformation and integrated care, was developed to structure the interviews. The topic guide was designed to encourage participants to reflect on factors shaping the implementation of NCMs from (i) a policy, (ii) organisational and (iii) local practice perspective. Transcribed interview data and fieldwork notes were analysed using thematic analysis to generate category systems and repeated themes ( Boyatzis, 1998 ). Drawing upon an interpretative approach, themes were developed iteratively and inductively, breaking down and reassembling the data through a coding process. To ensure analytical rigour, two members of the research team independently coded and analysed the qualitative data. These were then reviewed and discussed at wider research team meetings, with any discrepancies resolved through this process. Following the analysis within each site, a comparative case study approach ( Ragin and Becker, 1992 ) was used to compare and contrast factors shaping the implementation arrangements across all five NCMs. For confidentiality, all participants have been anonymised.

## Results

In the following sections, we explore factors shaping the implementation of five NCM pilot sites as they engaged with the multiple levels of context ranging from the macro policy level and the organisational responses at a meso level to the micro-level setting of workforce redesign. Although there is some analysis of each pilot site, our primary focus is on common issues and concerns evident across all five models. Unless otherwise stated, the quotations used reflect the general thoughts and views expressed by our interviewees.

### Negotiating uncertainty around policy and government targets

Our findings demonstrated that each pilot site had different aims and purposes, local arrangements and practices. These factors had to be set against a wider context of significant financial tensions, uncertainty around the direction of policy and fundamental questions about the future including the impact of more recent policy developments that, as noted earlier, are dominating the agenda. From a policy perspective, given the rapid pace of policy development in the NHS ( Hunter *et al.* , 2015 ; Jones *et al.* , 2013 ; Checkland *et al.* , 2012 ; Powell *et al.* , 2011 ), some interviewees felt that actions or changes imposed by government can negatively affect progress.

I think we've had so many central directive changes over the last 18 months that it really hasn't helped with trying to get buy-in. From new care models becoming very much NHS- driven programmes, to Sustainability and Transformation Partnerships superseding local plans, to various things that just create layer upon layer of uncertainty, really - a lot of goal-post changes. (EHCH-Senior Manager 6, CCG)

For some participants, the pace of such change is a particular concern. The following comment is typical:

I think one of the challenges has been the speed that we've had to go; the pressure that we've had from NHS England, because of being part of a national programme. I think that the speed and the pressure have suited other work streams, rather than it has mine. (EHCH-Senior Manager 10, CCG)

In this context, it was felt that government's pressure to deliver efficiencies and an undue emphasis on performance can hinder progress:

We've been influenced heavily though by the national direction of travel around standards and improvements and national must-dos, which at times has conflicted with what we've been attempting to do. (UEC-Senior Manager 5, CCG)

Many commentators stress the extraordinary pressures on the NHS to meet government targets and releasing efficiencies ( Hunter, 2006 ; Bevan and Hamblin, 2009 ; Bevan and Hood, 2006 ). Whilst some argue that market-driven reforms are a result of pressure on government by commercial bodies seeking security of income and healthy profits ( Leys and Player, 2011 ; Pollock, 2005 ), others highlight the extent to which market reforms in the NHS are outcomes of strong commitments to neoliberal values and beliefs, in particular the logic of markets and the principles of competition ( Pownall, 2013 ; Hudson, 2010 ; Reynolds *et al.* , 2012 ).

Overall, uniting all five pilot sites was their perception of the wider context within which they operated. They were critical in various ways of NHS England, particularly in terms of the unrealistic pressure placed upon them to deliver outcomes. There was a sense in which the pressure being felt was forcing the pilot sites to deliver without the appropriate substantive change being in place or sufficiently embedded and without being able to show sufficient or adequate evidence to support change. In this context, pressure for quick results was a major complaint:

There's been a lot of pressure from NHS England for certain things to be done on frameworks and time series and delivery plan sort of thing, so there is often a push from the office-based vanguard staff that we need to get certain things done. A clinician always puts the patient first whereas a project manager puts the project first, so that can be quite difficult. (EHCH, Senior Manager 14, CCG)

Of particular concern was the sheer scale and pace of change at the same time as the NHS was being tasked with making significant, if unrealistic, efficiency savings. Reflecting on the current fast-paced policy environment, interviewees in all five pilot sites criticised NHS England for failing to appreciate the length of time “change” takes. This was particularly the case in that the process was largely iterative and there needed to be appropriate measurement and monitoring of progress. As a participant in the ACC pilot commented:

I think the biggest gap is around understanding effective measurement and recording of progress. I think because of that gap, we don't understand if we are getting better or not, and we don't monitor and effectively embrace change. (ACC-Senior Manager 2, CCG)

### Legitimating return on investment and measuring performance

A number of interviewees pointed to the benefits of being able to draw upon the support from the national programme, but there was evidence of a tension between national pressures and the need to maintain locally driven change ( Ham, 2014 ). As a participant in the MCP pilot commented:

So the demand to see efficiencies to deliver…feels very top-down from a very high level…particularly in the last year as opposed to the few years before that when we've had time to do a bottom-up drive for designing change. (MCP-Senior Manager 2, CCG)

At the same time, although interviewees believed that hitherto national policy and the NCM programme had been an advantage, others spontaneously spoke of their fear that the “ *rug would be pulled* ” from under them with, for example, one model being recommended over all of the others or alternatively, there would be a major change in policy.

From a national driver point of view, what's around the corner? What's going to be the flavour of the month? So we put all this effort into the Five Year View, New Care Models and I think that for me is the main thing. (MCP-Senior Manager 4, CCG)

In this context, discussions regarding the national (i.e. English) NHS agenda tended to fall broadly into a number of categories. There was a minority group of respondents who acknowledged the invaluable support they believed they had received through being part of the NCM programme. For most, however, this clearly was thought to have come at a price. As one respondent in the PACS pilot commented:

There's an incredible level of scrutiny on you to be successful. I think the politics of it play out in the sense of trying to give you enough time to see results but at the same time, wanting results really fast so that they can roll models out nationally…it worries me we get the right answers. (PACS-Senior Manager 3, CCG)

In this context, a number of interviewees criticised the NCM programme's ambitious plans for sustainable transformation during a period of significant financial pressures and uncertainty for the future of the NHS ( Alderwick and Ham, 2017 ). Within all pilot sites, there were concerns that too much was being expected too soon in terms of demonstrating a “return on investment” in digital capacity. It was felt that digital innovation is almost always a complex undertaking due to the rapid advancement of digital technology and fast-paced change.

Nothing really gets time to bed in before the next initiative comes along – they give you £1m and want to know the return on investment is £1.0325!. (UEC-Senior IT Manager 2, CCG)

Availability of resources was considered to be a key factor for the successful implementation of each NCM. However, uncertainty around the availability of funding was evident within all sites. For example, cuts in the anticipated funding to digital developments have already made an impact:

Funding for urgent and emergency care is absolutely off the table. It has allowed us to get to this point but there is a lot more that could be done (UEC-Senior IT Manager 1, CCG)

### Managing different structures and governance arrangements across care settings

At an organisational level, although participants felt that the NCM initiatives have the potential to address the problem of silo working across organisations, they also acknowledged that current organisational arrangements could sometimes be a barrier to successful joint working. As one interviewee in the Care Home pilot commented:

At the moment, there's a boundary line that comes in between each thing that you do. “That's health. That's social work.” It shouldn't be like that. It should be everybody working together for one outcome for the patient or the service user. (EHCH-Senior Manager 7, CCG)

In this context, it was felt that different organisational structural and governance arrangements across different providers could serve as a barrier to the delivery of the programme's aims and objectives ( Degeling *et al.* , 2004 ). As an interviewee in the UEC pilot commented:

We have two acute trusts and the focus in each acute trust is very different, and the pressures in each acute trust are very different, and they conflict. (UEC-Senior Manager 3, CCG)

Although interviewees reported how successfully relationships had been developed with different sectors, a central focal point of discussions concerned the difficulties that the work and nature of the NCMs could cause with external partners. For example, in the case of the ACC pilot, the innate competitiveness of hospital trusts ran somewhat counter to acute care collaboration and at times was thought to harbour suspicion and mistrust.

Then, there needs to be a bit of a behavioural shift, because by nature hospital trusts are competitive with each other and counter to the collaborative approach, which is what acute care collaboration is about. Generally, it can be quite parochial. (ACC-Senior Manager 1, CCG)

Financial constraints were also thought by respondents to reinforce organisational protectionism ( Dixon-Woods *et al.* , 2014 ) which in turn discouraged or prohibited collaborative working with external partners and further encouraged the fragmented nature of the system.

It had been harder convincing potential partners that the relationship would be built upon collaboration and not competition or indeed acquisition. In this regard, difficulties were highlighted but most felt that lessons had been adequately learned. The following view is typical of those expressed in interviews.

I think it is going back to prior to the Vanguard we were going through a process to acquire xxx. I think that learning has helped us to understand some unintended consequences that we wouldn't want to repeat around culture, and how during major change cultures collide, and what we would do differently. (ACC-Senior Manager 1, CCG)

More complex though was the balancing of collaborating with different partners within different sectors particularly since the concept was clearly not aligned with the profit-making motives of commercially based organisations.

As soon as you start developing models with organisations such as Rovera, PWC, Metronics, they have a slightly different incentive. So ensuring we have the right balance and we keep patients at the heart of it, which is what we hope to achieve to do, but you can absolutely understand that will always be a challenge to get to the right place. (ACC-Senior Manager 7, CCG)

### Reconfiguring inter-organisational relations and practices

The development of multi-disciplinary teams (MDT) to facilitate successful implementation of each NCM pilot was viewed as one of the most important benefits across all sites ( Carter *et al.* , 2003 ). Sharing of aims and learning about the viewpoints of others was felt to help with this, along with the growing recognition that joint working was the only way to work in times of severe budget constraints and cuts. Although the multi-agency, multi-disciplinary input and the ability to bring in other experts were valued, it was felt that there could be problems when new organisations, or new representatives, came along, in terms of bringing them up-to-date with the intentions and progress of the NCM programme. For some participants the inclusion of many different organisations could also add complexity.

You're pulling together lots of different employers and areas of work which, although all the people in the room might be very up for all working together, once you bring the bigger beasts in, it's not as simple as that … you're wrestling, then, with lots of different sets of values, ability to change, flexibility… (EHCH-Senior Manager 5, CCG)

Despite these challenges, relationship building between care settings had clearly been a major feature of all the NCMs, and there was overall a sense in which there had been improvements at different levels. For example, it was felt that communication and relationship building were continuing to improve between care settings although it was suggested by a number of participants that the level of trust since the inception of Payments by Results (PbR) (the system in NHS England under which commissioners pay healthcare providers for each patient treated, taking into account the complexity of the patient's healthcare needs) and the commissioner–provider split had been a fundamental barrier.

In terms of partnership working, it was notable during discussions that on the basis of the complexity of the changing matrix of relationships, it was apparent that respondents found it difficult to focus upon the nature of and changes in inter-professional communication. This was made explicit by one interviewee:

I think if you can create some relationships and friendships way before you start any project you always going to do better than just launching straight in. I think creating the right system and the right relationships make it work…it is essentially then understanding and emphasising with your colleagues and actually looking at where the ability for joint working is. (ACC-Senior Manager 6, CCG)

Even though relationships between health and social care had been built up over many years, it was thought they had not really materialised on the ground. One respondent reported that the contrast between working within the “flat structure” of the Clinical Commissioning Group and the bureaucratic and hierarchical structure of the Foundation Trust and local authority was particularly challenging. Notwithstanding this, many interviewees did comment upon the long-established working partnership between health and social care encouraged by co-location, but for some this had not necessarily enabled a greater understanding.

So the people who would be my equivalent colleagues, we don't spend any time together - we don't really understand what each other is doing and whether there is any crossover or conflict. (PACS-Senior Manager 4, CCG)

Difficulties in operational relationships were also evident between the acute and community sectors and the seeming lack of enthusiasm among acute clinicians for working in the community.

We still haven't cracked the relationship and models of care about how we pull our secondary care colleagues out working into the community more. We done some decent pilots of it at a local level, for example in xxx but what we haven't done is starting looking at that integration of relationships across the whole county that wraps around that. (PACS-Senior Manager 3, CCG)

Although there were concerns that inter-professional communication and understanding remained a challenge generally, it was felt by many that there was evidence that this was indeed shifting.

I think we are conquering quite a lot of the organisational stuff, but what we haven't landed yet is the culture and mindsets of how clinicians can work together in different ways - we have pockets of it in primary care, it's brilliant. (PACS-Senior Manager 2, CCG)

### Building capacity and resources

Participants valued the national programme for the “pump priming” that had allowed plans to get underway and be supported earlier than perhaps would have happened otherwise. Financial resources provided to the UEC pilot were valued by some interviewees:

It brings some extra capacity into the system to actually do some of the work. Obviously in the past they've also had financial resource, which I think has helped them gain traction and gain influence. I think of pieces of work, like the programme to roll out direct booking for GP practices via NHS 111, I don't think that would have happened without the financial resource that the Vanguard was able to bring to bear. (UEC-Senior Manager 2, CCG)

However, for many of those interviewed, implementing the NCM pilots was not, as they described, part of their “day job”, and for some this created an almost irreconcilable tension. As participants in the ACC pilot commented:

My role is not the Vanguard …and that's sometimes the tension because the day job is the day job….but everybody is incredibly pressurised and if you don't keep an eye on the day job, the day job gets worse. (ACC-Senior Manager 2, CCG)

Many of the interviewees were highly critical of the reduction in the programme's financial support and that there had been no guarantee of funding over the three years.

Sometimes to do a transformational change you do need a bit of pump priming - when you are getting half the funding that you need, that is a bit difficult…I think the challenges are the initial excitement has been a bit tampered down because of the way that the funding allocations were cut in subsequent years. (ACC-Senior Manager 1, CCG)

There was additionally a common perception that the short-term investment was insufficient to sustain the work and development and that once the financial support disappeared, the programme would continue but that the pace would be a good deal slower.

I am not confident with it coming to a sudden end…because if they are not providing any money or any funds how are they going to keep up the impetus on delivery? I don't think we'd stop because we've got that relationship with organisations now - I just don't know if it would continue as extensively as it is doing now. (ACC-Senior Manager 4, CCG)

Aside from resources, time and “back-fill” of staff were additionally considered to be major barriers ( Exworthy *et al.* , 2010 ; Dixon-Woods *et al.* , 2014 ). Further, staff had to see the value and benefit of the team.

I think the biggest issue about MDT working is creating the time where people I think are working exceptionally hard. There isn't an additional workforce that you can put in because there is nobody to back-fill…it is less about the money and more about the workforce. (PACS-Senior Manager 1, CCG)

With regard to protected time for meetings, those professionals whose time was funded (so that they could get cover for sessions) felt this allowed them to attend MDT meetings and participate to a greater extent. As a participant at the Care Homes pilot commented:

One of the benefits is having the time to think about what is useful. Normally as a GP you don't get much time to reflect on the value of what you are doing or why you are doing it, or how you might be doing it. (EHCH-Senior Manager 12, CCG)

However, there appeared to be some resentment that not everyone's time was covered and that for many the tasks undertaken and meetings attended were just assumed to be part of their everyday responsibilities. As a participant at the Care Homes programme commented:

Why was it allowable to pay for GP and consultant time, but not to give something to care homes for their involvement? (EHCH-Senior Manager 6, CCG)

### Securing commitment and engagement

Amongst all pilot sites, there was much praise for the very high levels of commitment shown by participants. This was felt to lead to much better outcomes, with people keen to meet objectives and to share experiences or learning ( Erskine *et al.* , 2013 ). As one interviewee in the Care Homes pilot commented:

Everyone in there wants to be there, I think and wants to make a difference and wants to work on behalf of their organisations to start building bridges and improving systems. (EHCH-Senior Manager 1, CCG)

In this context, buy-in from organisations or particular professional groups was also considered key to success but often a very challenging task. As one participant in the Urgent and Emergency Care pilot commented:

I think what helps the Vanguard project is the buy-in … getting some of the understanding and the buy-in from some of our local authority partners, has been very challenging. (UEC-Senior Manager 7, CCG)

Although, there was thought to be a lot of committed people within the region, interviewees noted that not all providers had fully signed up to working within the NCM programme. In particular, concerns were raised in the PACS pilot that some Trusts had not yet agreed to participate to the ACO leading one interviewee to comment as follows:

The elephant in the room is the fact that we have a great big hospital trust which still sits in the area…It is a bit of a concern because from a needs perspective the people that go to that hospital tend to be more affluent…we are just going, oh that's a bit hard, let's concentrate on the easy stuff, rather than looking at the whole thing. (PACS-Senior Manager 4, CCG)

Some argued that the programme had been left to key individuals and although other members of staff were kept informed, there was a perception that the understanding had not filtered through into the wider healthcare system. It was hard to make the necessary and at-speed change when full collective ownership was not present. Again, attention was drawn to the perceived isolated pieces of work and accompanying lack of awareness.

I mean the challenge, which we think we crack but we don't really crack is engagement. Engaging health care workers and other leaders in the system…I would say it is a fragile thing, engagement from leaders to healthcare workers, particularly GPs, it has to be developed. (PACS-Senior Manager 9, CCG)

Although opinions were divided as to the interplay between organisational and personal relationships, the latter were an undeniable factor. Some explained how clashes of personalities had been quite instrumental in subsequent organisational development. As one participant in the PACS pilot commented:

I think the key relationships were very strained at the beginning…there was a clash of personalities, a clash of priorities and just a clash of focus. (PACS-Senior Manager 8, CCG)

## Discussion

Drawing upon recent theoretical developments on the multilevel nature of context ( Maniatopoulos *et al.* , 2015 ; Greenhalgh *et al.* , 2017 ), this paper has explored factors shaping the implementation of five NCMs in the North East of England. Despite the *5YFV* 's emphasis on “local flexibility” ( NHS England, 2014 , p. 4) to support implementation, our findings demonstrate that all five pilot sites experienced, and were subject to, unrealistic pressure placed upon them to deliver outcomes. There was a sense in which the pressure coming from the centre (i.e. NHS England) and being felt was forcing the pilot sites to deliver without the appropriate substantive change being in place or sufficiently embedded and without there being adequate reliable evidence to support change. In particular, there was a perception that government targets and an undue emphasis on performance were seriously hindering progress ( Hunter, 2006 ; Bevan and Hamblin, 2009 ; Bevan and Hood, 2006 ). The overriding impression, particularly in the PACS pilot, was that there were pockets of excellence and impressive examples of new working, but this was not replicated evenly or consistently across the programme as a whole. There was, though, some evidence emerging in terms of the development of local hubs or federations of GPs which were thought to be sustainable. Of particular concern among all pilot sites was the sheer scale and pace of change occurring at the same time as the NHS was being tasked with making significant, if unrealistic, efficiency savings.

At an organisational level, the need for, and importance of, relationship building was also common to all five sites, but in each there appeared to be different obstacles to progress ( Hunter *et al.* , 2015 ). It was suggested that the national programme helped individual sites to build inter- and intra-organisational relationships. Nonetheless, common to all five was the significant amount of effort and time that had been put into creating better relationships among partners. The need to ensure adequate support across, and within, the relative organisations, employing both personal connections and available structures in varying degrees, was another common theme across all pilot sites. Some respondents across the sites struggled with the concept of networks, either informal or formal, seemingly on the grounds that they were either simply unaware of their existence or because such networks were not perceived to be particularly relevant to the NCM programme. Some participants (e.g. in the PACS pilot) felt, however, that there was definitely a perception that networks were being driven more purposefully.

In all sites participants felt that the national programme helped to raise the profile of local change initiatives and also contributed to the wider understanding of regional service integration issues. Moreover, it was felt that the programme enhanced or speeded up certain actions (in particular regional MDT involvement). However, the need for a system-wide approach was recognised and an emphasis was placed on collective rather than individual action ( Hunter *et al.* , 2015 ). In the UEC pilot, it was felt that there were economies of scale to be achieved because of the way the ambulance service covered a much larger area than an individual CCG. In both the PACS and the MCP pilots, there was very much a sense that this was the only way to go given the financial difficulties which existed, but this did not detract from a genuine belief that it was also the right way to proceed in terms of patient care. Indeed, there was criticism, especially in the MCP pilot, that certainly underlaid some of the discussions that the programme should have been more “system and patient driven” rather than “cost” driven. Similarly, in the ACC pilot, a system-wide approach was recognised with an emphasis on the need for long-term investment required to sustain the work and development of the NCM. In all sites there were tensions between the need for real investment in terms of capacity, capability and finance, the accompanying risk and the ability to deliver outcomes. In particular, concerns were raised over the lack of additional resources to support transformation efforts within each pilot site.

There were varied opinions amongst the interviewees about the value of MDT meetings. In the EHCH pilot, it was felt that a sharing of aims and learning about the viewpoints of others was helping to combat the silo working for which health and social services have long been accused and often found guilty, alongside a growing recognition that joint working was the only way to operate during times of severe budget constraints and cuts ( Hunter, 2016 ; Degeling *et al.* , 2004 ). However, there was also a feeling that the inclusion of many different organisations could add further complexity. Similarly, in the UEC pilot, it was felt that the differences in perspective among the various organisations, related political agendas and the differences in governance structures across organisations could pose barriers. Trust between agencies was considered to be essential but, at the same time, it was recognised that good relationships can take time to develop. MDT working was seen by both the PACS and MCP pilots as something which needed clear thought with respect to purpose, stratification of patients and appropriate management. In both pilots there was also a need to “rethink” skill mix. The experience of using community pharmacists in the PACS pilot was seen by almost all as a huge success and an example of how skill mix might work. Whereas in the MCP the main barrier appeared to be moving to one organisation, in the PACS it was the importance of scaling up the pilots but without losing the local nuance.

This exploratory study of five local initiatives over a short period of time provides an initial mapping of the ongoing implementation of the NCMs. Our analysis advances the study of large-scale health system implementation in three ways. First, it broadens the scope and scale of analysis across the multiple levels of context that contributed towards the successful implementation of large-scale health system change initiatives ( Maniatopoulos *et al.* , 2015 ; Greenhalgh *et al.* , 2017 , 2016 ; Robert *et al.* , 2010 ). In this study we have not examined those factors shaping the implementation of NCMs in a particular context but rather have explored the interdependent relationships between different structural elements of the context across a multilevel set of practices, ranging from the macro policy level and the organisational responses at a meso level to the micro-level setting of individual action/workforce redesign. In so doing, our findings reinforce the importance of moving beyond the boundaries of local practice towards a broader perspective of place-based interconnected practices shaping policy implementation and change ( Maniatopoulos *et al.* , 2015 ).

Second, our theoretical positioning stresses the importance of being sensitive to the broader political and ideological context of policy implementation in which large-scale health system transformation takes place ( Hunter *et al.* , 2015 ; Navarro, 2011 ; Jones, 2017 ). Health system change initiatives in themselves are not neutral; they reflect particular political values, beliefs and ideologies although these underlying political assumptions are never specified by their proponents. In this context, achieving whole system change is particularly vulnerable to the vicissitudes of politics especially where that system, like the UK NHS, is itself subject to those very same pressures. Our findings demonstrate how the implementation of the NCMs in England is framed within a climate shaped by a strong and persistent audit culture, new public management practices and a neoliberal set of ideas with an emphasis on the delivery of quick efficiency savings, on measuring performance and on the containment of public spending on services like the NHS. Within all the pilot sites there were concerns that government targets dominated the activities of local initiatives and displaced objectives such as the opportunity for shared learning. Much of the extant literature around health systems implementation has decontextualised it from the wider setting and focused on the structural elements of the systems, or on the processes and relationships between elements of the systems, rather than exploring the surrounding political environment within which the changes being implemented are taking place ( Hunter *et al.* , 2015 ).

Finally, this study seeks to move beyond the single-site implementation of health policy by exploring the implementation of the NCM programme within a wide range of organisations and related stakeholders involved in the delivery of the NHS strategy. Single-site implementation studies provide an invaluable tool for studying in a vertical way the impact of health policy on practice and have enabled the assembly of rich and detailed local ethnographies of the adaptive response by single organisations to the translation of policy into practice. However, to view large-scale health system transformation principally at the point where a single organisation encounters it risks missing the wider context of policy implementation which in turn could lead to somewhat partial understandings of these complex processes. In particular, it downplays important influences/interdependencies between organisations, settings and practices. We argue that if we are to understand the full implications of large-scale health system change initiatives, then we should study how health policy implementation is played out over multiple time frames, organisations and settings. To understand its shaping therefore requires researchers to go beyond the study of policy implementation at a single locale or moment and, rather, attempt to follow it through space and time. Given that complex change represents a continuous journey rather than something that happens at a single defined point in time, there is great merit in seeking to evaluate such developments over time taking into account the myriad factors and pressures evident in the wider context.

In sum, our findings have demonstrated the need for a deeper understanding of the multilevel nature of context by exploring factors shaping the implementation of five NCMs in the North East of England. Exploring the wider national policy context is desirable as well as understanding the perceptions of front-line staff and service users in order to establish the degree of alignment or, conversely, to identify where policy and practice are at risk of pushing and pulling against each other. Furthermore, in a context where devolution is a live and evolving issue in England, research is needed to examine and understand the current implementation of the NCM programme with a view to establishing how far, if at all, the regional dimension is a significant factor in transformation efforts and one perhaps meriting additional support and attention.

## Conclusions

This study was conducted within a limited time period during which there has been considerable and continuing policy churn, notably developments surrounding STPs and ICSs, and more recently ICPs, accompanied by growing financial pressures on the NHS despite an improved funding path for the NHS averaging 3.4 per cent over the next five years compared with 2.2 per cent over the past five years. Inevitably, this has raised issues and concerns about the sustainability of the positive developments underway across the NCM national programme which have been identified and some of which we have drawn attention to in this paper. Ensuring that the changes have a chance of becoming embedded and sustainable over time requires reconfigurations across a multilevel set of practices as well as a continuing commitment politically to invest in support and development alongside creating and protecting the space to enable change to occur and prosper.

It is far too early to conclude with any confidence that a successful outcome for the NCM programme will be forthcoming although the NHS Long-Term Plan seeks to build on the earlier vision set out in the Five-Year Forward View. Early indications show some signs of promise, especially where there is evidence of the ground having been prepared and changes already being put in place prior to the official launch of NCM initiatives. But the overall context(s) in which the complex and ambitious changes are being implemented remains both fragile, if not febrile, and fluid. Managing such a context(s) as the NHS Long-Term Plan gets implemented over the next few years will be critical if the changes are going to survive and yield the desired impact on health and well-being for the population in the North East of England.

## Figures and Tables

**Table I:** “Receptive contexts for change” framework

Factors	Description	Focus of analysis	Empirical focus
Quality and coherence of policy	Evidence-based policy and alignment between goals, feasibility and implementation requirements at both national and local levels	Macro level	The role of policy priorities for improving health care
Environmental pressure	Environmental conditions for transformational change, including political context	Macro level	The role of financial and public concerns to service improvement
Supportive organisational culture	Organisational support and commitment for change	Meso level	The role of shared values, beliefs and patterns of behaviour
Change agenda and its locale	Local political culture and support for organisational changes	Meso level	The role of local pressures to improve health services
Simplicity and clarity of goals and priorities	Identified goals, priorities and a pathway for change that is sustainable over time	Meso level	The role of multiple priorities at both national and local levels
Cooperative inter-organisation network	Joined-up thinking/partnership arrangements across organisational and professional boundaries – formal and informal	Meso level	The role of partnerships, alliances or other collaborations between professionals and organisations working together
Managerial–clinical relations	High-trust managerial–clinical relationships	Micro level	The role of relationship building through multi-disciplinary teams
Key people leading change	Leadership and adoption of a shared vision, values and beliefs	Micro level	The role of credible change agents/champions to lead change/system leadership

**Table II:** List of interviewees

Vanguard	No. of interviews	Interviewees
MCP vanguard	7	Senior manager, CCG
MCP vanguard	1	Senior manager, LA
MCP vanguard	3	Senior IT manager, CCG
PACS vanguard	11	Senior manager, CCG
PACS vanguard	2	Senior IT manager, CCG
ACC vanguard	7	Senior manager, CCG
ACC vanguard	3	Senior IT manager, CCG
Enhanced health in care homes vanguard	14	Senior manager, CCG
Enhanced health in care homes vanguard	3	Senior IT manager, CCG
Urgent and emergency care vanguard	11	Senior manager, CCG
Urgent and emergency care vanguard	4	Senior IT manager, CCG
*Total*	*66*	

**Table AI:** Vanguard sites

Vanguard	Aim of programme
Multi-specialty Community Providers (MCPs)	The Vanguard aims to move care out of the hospital into the community. It involved the implementation of an out-of-hospital model of care focusing on: people staying independent and well for as long as possible; people living longer with a better quality of life with long-term conditions; people supported to recover from episodes of ill health and following injury; resilient communities and high levels of public satisfaction. The MCP Vanguard began in April 2015 although pre-Vanguard elements began implementation from 2013
Primary and Acute Care Systems (PACS)	The Vanguard aims to develop a new variant of “vertically integrated” care allowing single organisations to provide joined-up GP, hospital, community and mental health services. It involved the development of a new Urgent and Emergency Care Hospital and the development of an “enhanced care teams” pilot and new workforce models (Transforming Primary Care). The PACS Vanguard began in June 2015 and the Trust became the first Accountable Care Organisation in the region – effective from April 2017
Acute Care Collaboration Vanguard (ACC)	The Vanguard aims to link local hospitals together to improve their clinical and financial viability, reducing variation in care and efficiency. It aims to widen the support and services (i.e. commercial/contractual services, consultancy/advisory as well as a range of clinical and corporate services) the Trust can provide to other parts of the NHS through acquiring and/or merging with other hospital Trusts. The ACC Vanguard was finalised in January 2016
Enhanced Health in Care Homes Vanguard (EHCH)	The Vanguard aims to offer older people better, joined-up health, care and rehabilitation services. It aims to develop a sustainable, high-quality new care model for people in community beds and receiving home-based care services across a metropolitan area with a new outcome-based contract and payment system that supports the development of the Provider Alliance Network (PAN) delivery vehicle. The Vanguard started March 2015 although some features had been implemented pre-Vanguard status
Urgent and Emergency Care Vanguard (UEC)	The Vanguard aims to improve the coordination of urgent and emergency care as a whole system, ensuring people can access the most appropriate service, first time. The Vanguard status was awarded in July 2015 and the programme has been fully operational since November 2016. Most initiatives went live in December 2016

## Glossary

ACC: Acute Care Collaboration Vanguard
CCG: Clinical Commissioning Group
EHCH: Enhanced Health in Care Homes
GP: General Practice
ICPs: Integrated Care Partnerships
ICSs: Integrated Care Systems
MCPs: Multi-speciality Community Providers
MDT: Multi-disciplinary Team
NCMs: New Care Models
NHS: National Health System
PACS: Primary and Acute Care Systems
PbR: Payments by Results
STPs: Sustainability and Transformation Partnerships
UEC: Urgent and Emergency Care
UK: United Kingdom
5YFV: NHS Five-Year Forward View

## Appendix

Appendix A
